# Impacts of selenium and vitamin E supplementation on mRNA of heat shock proteins, selenoproteins and antioxidants in broilers exposed to high temperature

**DOI:** 10.1186/s13568-018-0641-0

**Published:** 2018-07-10

**Authors:** Shahnawaz Kumbhar, Alam Z. Khan, Fahmida Parveen, Zaheer A. Nizamani, Farman A. Siyal, Mohamed E. Abd El-Hack, Fang Gan, Yunhuan Liu, Muhammad Hamid, Sonia A. Nido, Kehe Huang

**Affiliations:** 1 Institute of Nutritional and Metabolic Disorders in Domestic Animals and Fowl, College of Veterinary Medicine, Nanjing Agricultural University, Nanjing, 210095 Jiangsu China; 2 Department of Veterinary Pathology, Faculty of Animal Husbandry and Veterinary Sciences, Sindh Agriculture University, Tandojam, 70060 Pakistan; 3 Department of Animal Nutrition, Faculty of Animal Husbandry and Veterinary Sciences, Sindh Agriculture University, Tandojam, 70060 Pakistan; 4 Department of Poultry, Faculty of Agriculture, Zagazig University, Zagazig, 44511 Egypt

**Keywords:** Broiler meat, Selenium, Vitamin E, Antioxidant capacity, Selenoproteins, Heat shock proteins

## Abstract

The study was carried out to investigate the effect of dietary selenium (Se) and vitamin E (VE) supplementation on mRNA level of heat shock proteins, selenoproteins, and antioxidant enzyme activities in the breast meat of broilers under summer heat stress conditions. A total of 200 male broilers (Ross 308) of 1 day age were randomly separated into 4 groups in a complete randomized design and were given a basal diet (Control, 0.08 mg Se/kg diet) or basal diet supplemented with VE (250 mg/kg VE), sodium selenite (0.2 mg/kg Se), or Se + VE (0.2 mg/kg Se + 250 mg/kg VE) to investigate the expression of key antioxidant and heat shock protein (HSP) genes under high temperature stress. Dietary Se, VE and Se + VE significantly enhanced the activities and mRNA levels of catalase as well as superoxide dismutase (SOD) but decreased the mRNA levels of *HSP70* and *HSP90*. Se alone or combined with VE increased the concentration of selenoprotein P and selenoproteins mRNA level and decreased the expression of *HSP60*. In addition, Se and Se + VE significantly enhanced the glutathione peroxidase (GPx) activity and the expression of *GPx1* and *GPx4* in breast muscle tissues. It is noteworthy that all the treatments significantly decreased malondialdehyde (MDA) level in the breast meat. Overall results showed that Se in combination with VE has maximal effects to mitigate heat stress. Based on given results it can be recommended that Se + VE are a suitable dietary supplement for broilers to ameliorate the negative effects of summer heat stress conditions.

## Introduction

Heat stress affects the performance of the birds (Gregory 2010). Previous studies showed that meat productivity decreased in chronic heat stress conditions (Hashizawa et al. 2013). The high ambient temperature changes the metabolism and body composition of chickens (Zeferino et al. 2016). The heat stress increases mortality, decreases feed intake and feed conversion efficiency, reduces body weight gain thus low carcass weight, and decreases meat quality in the broiler chickens (Wang et al. 2009; Zhang et al. 2012; Alagawany et al. 2017). Moreover, it causes a decrease in the vitamin (E and A) and mineral (selenium, iron and zinc) concentrations of tissue (Sahin and Kucuk 2003). The reduced iron, zinc, and selenium levels result in reduced oxidative capacity (Kelman et al. 2014). The heat stress induced changes (biochemical as well as physiological) potentially enhances the formation of reactive oxygen species (ROS) (Mujahid et al. 2007; El-Kholy et al. 2017, 2018) which in turn disturb the balance of oxidation as well as antioxidant defense systems to induce lipid peroxidation and cause oxidative damages to biological molecules including proteins and DNA (Lin et al. 2006). Broilers kept under acute heat stress manifested more than a twofold increase in malondialdehyde content of breast meat, which is a secondary product of lipid oxidation (Mujahid et al. 2009).

All cells and tissues produce higher levels of *HSPs* under high temperatures. Stress inducible proteins like *HSP70* work as molecular chaperons that safe guard cells and organisms as they keep cellular proteins in a competent folding condition to prevent the aggregation of irreversible proteins and help in the refolding of proteins damaged by the stress (Gabriel et al. 2001). While the main role of *HSPs* is to prevent and reverse the damage to proteins, the antioxidant enzymes decrease oxidation and prevent oxidative damage. Hyperthermia produces oxidative stress and increases the production of ROS (Robert et al. 2017) which results in the induction of the expression of *HSP70* (Zhang et al. 2006). The major role of antioxidants is to reduce the free radicals and prevent the lipid peroxidation that protects cells from ROS (Nanari et al. 2004; Grashorn, 2007). The antioxidants are either enzymatic or non-enzymatic. Superoxide dismutase and glutathione peroxidase are enzymatic antioxidants whereas, lipid-soluble vitamins (E and A), pro-vitamin A (beta-carotene) and water-soluble vitamin C are non-enzymatic antioxidants. Low plasma concentration of antioxidant vitamins (C and E, and folic acid) and minerals (Se and Zn) are negatively correlated with high damage caused by oxidation in poultry reared under stress conditions (Sahin et al. 2002).

Selenium, an essential trace element, is regarded as an integral component of Se-dependent glutathione peroxidase (Yoon et al. 2007), and when accompanied by VE forms a part of the cell defense system against free radicals (Surai 2002). The GPx mainly eliminates the extra quantities of peroxides as well as hydrogen peroxide in fatty acids produced due to the oxidation of lipids (De Almeida et al. 2012). Previously reported that deficiency of VE, Se or both, disturbed the immune system of young chickens (Swain et al. 2000). Moreover, Se and VE act synergistically (Bou et al. 2001). Requirement of VE increases when Se is deficient (De Almeida et al. 2012). Therefore, particular emphasis has been placed on selenium as well as on VE, which are the two important factors for improving the health and the performance of the birds and their meat quality (Ševčíková et al. 2006). In addition, broiler diet supplemented with 0.84 mg/kg of Se (as selenium-yeast) resulted in the increased Se concentration of raw thigh muscle (Haug et al. 2007). Similarly, high Se level of the broiler diets resulted in increased selenium concentration in breast meat, which is responsible for higher oxidative stability of lipids (Ševčíková et al. 2006). Moreover, Se deficient chicken diet affected the immune organs, decreased serum interleukin-1β, interleukin-2 and the serum tumor necrosis factor, which indicates that oxidative stress restricts immune organs development, thus disturbing the immune system of chicken (Zhang et al. 2005). Previous study reported that Se deficiency decreased the messenger RNA levels of *Gpx1*, *Gpx4*, and *Sepp1* in the visceral adipose tissues of chicken (Liang et al. 2014).

Previous investigations showed a positive influence of VE or Se on the defense system of cells and subsequent prevention of heat stress in chickens when reared under controlled temperature conditions (Mahmoud et al. 2003). Whereas, very limited research has been conducted to investigate whether dietary antioxidants played their part in heat stress protein modulation as well as antioxidant level in broiler chickens reared in summer hot environment. The present research was conducted to investigate the effects of supplemented diet with Se, VE, or their combination on the endogenous antioxidant profile, selenoprotein expression and heat shock proteins in meat of broiler reared under high ambient temperature.

## Materials and methods

### Chemicals

The GSH-Px, SOD, catalase, MDA and the total protein assay kits were obtained from Nanjing Jiancheng Bioengineering Institute and Biyuntian Institute (Nanjing, China), respectively. Stock standard solutions for sodium selenite [GBW (E) 080215] as well as VE (α-tocopherol acetate) were supplied by the National Research Center for Standard Materials and Food Detection Science Institute (Beijing, China). Real-time PCR reagents were obtained from TaKaRa (Dalian, China).

### Animals and diets

Two hundred day-old male broilers (Ross 308) of 45 ± 0.5 gm average body weight were randomly selected and allotted to four treatments in a complete randomized design experiment, each with five replications (10 birds per replicate). The four diet treatments provided to chickens were: basal diet without any supplementation of VE or Se (Control group), basal diet + 250 mg of VE/kg diet (VE group), basal diet + 0.2 mg of Se/kg diet (Se group), and basal diet + 250 mg of VE/kg and 0.2 mg of Se/kg (VE + Se group). The basal diet composition (Table 1) applied was in accordance with the requirements proposed by National Research Council (NRC 1994). The basal diet comprised of 0.08 mg Se/kg of feed with 30 mg/kg of vitamin E. The birds were given starter diet up to day 21, followed by finisher diet up to 42 day of the age.

**Table 1:** Composition and nutrient levels of basal diet for broiler chickens (g/kg)

Ingredients	Starter	Finisher
Corn	59.1	64.3
Soybean meal	30.6	24.3
Corn gluten meal	3.80	4.50
Vegetable oil	1.70	2.50
Limestone	1.31	1.23
Dicalcium phosphate	1.77	1.58
Sodium chloride	0.42	0.33
l-Lysine	0.15	0.16
DL-methionine	0.15	0.10
Premix^a^	1.00	1.00
Calculated composition		
Metabolizable energy, MJ/kg	12.27	12.77
Crude protein (%)	21.2	19.3
Calcium (%)	1.00	0.91
Available phosphorus (%)	0.43	0.38
Lysine (%)	1.08	0.95
Methionine (%)	0.50	0.43
Methionine + cystine (%)	0.82	0.73

^a^Provided per kg of diet: iron, 60 mg; copper, 7.5 mg; zinc, 65 mg; manganese, 110 mg; iodine, 1.1 mg; bacitracin zinc, 30 mg; vitamin A, 4500 IU; vitamin D3, 1000 IU; vitamin E, 30 IU; vitamin K, 1.3 mg; vitamin B1,2.2 mg; vitamin B2, 10 mg; vitamin B3, 10 mg; choline, 400 mg; vitamin B5, 50 mg; vitamin B6, 4 mg; biotin, 0.04 mg; vitamin B11,1 mg; vitamin B12, 1.013 mg, Se, 0.08 mg/kg

### Husbandry

**Fig. 1:**
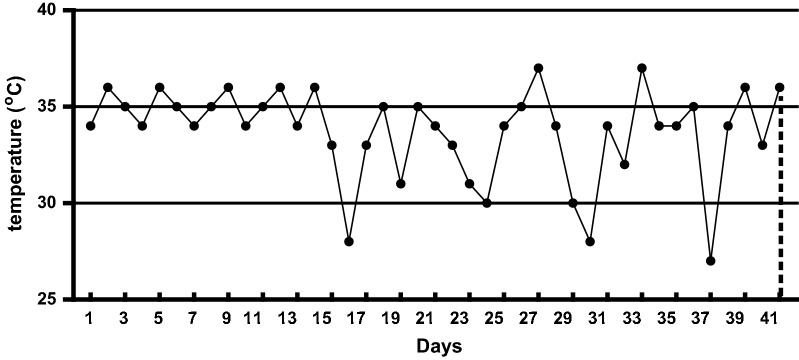
Average daily temperature during summer season from 12:00 am to 8:00 pm for 42 days of experiment

The experiment was conducted at Nanjing Agricultural University, Nanjing (32°0′N latitude, 118°47′E longitude), in the Jiangsu province of China. All the experiments conducted on the experimental animals were approved by the Ethical Committee of Nanjing Agricultural University, Nanjing P.R. China vide Animal Ethical Code number SYXK-Su 2011-0036. The brooding temperature inside broilers shed was environmentally controlled during the first 14 days of the experiment which ranged from 34 to 36 °C, and then the broilers were reared under the natural ambient temperature of summer conditions. The weather in summer during the last 28 days of the experiment had a typical East Asian climate, the average temperature ranging between 30 and 37 °C in the day and 25 and 29 °C in the night. The average daily natural ambient temperature was recorded (12:00 a.m. to 8:00 p.m.) during the experiment is depicted in Fig. 1. The daily relative humidity in the shed ranged from 60 to 80%. Light was provided for 24 h during the first 3 days followed by 23 h until day 7 and 18 h till the end of feeding trial. All birds were immunized against Newcastle disease and infectious bursal disease virus. The feed and water was provided ad libitum.

### Samples collection and processing

The forty broiler chickens (2 birds per replication) were randomly selected at the end of the experiment and slaughtered. Breast muscle samples were individually sliced in different sections. Two-fourth of breast muscle was excised and kept at − 20 °C for the antioxidant enzyme activities and MDA analysis. Similarly, for the analysis of mRNA levels, one-fourth of the breast muscle was excised rapidly and perfused with cold isotonic saline, then snap-frozen using liquid nitrogen and kept at − 70 °C.

### Determination of antioxidant enzyme activities and selenoprotein P (SelP) concentration

Breast muscle sample (1 g) was homogenized for 10 s at 8000 rpm in physiological saline on ice using Ultra-Turrax homogenizer (Tekmar Co., Cincinnati, OH). It was then centrifuged at 4000 rpm for 15 min at 4 °C. The supernatant thus obtained was utilized in further analyses. The GSH-Px, SOD, catalase as well as MDA were measured spectrophotometrically by using protocol of kits commercially available from Nanjing Jiancheng Bioengineering Institute, Nanjing, Jiangsu, China (Ahmad et al. 2012). The SelP concentration was measured using ELISA as described by Saito et al. (2001). Protein concentration was obtained using Bradford Protein Assay Kit (Beyotime Institute of Biotechnology, Jiangsu, China) whereas bovine serum albumin was used as the standard.

### RNA extractions, reverse transcriptions and real-time PCR

The total quantity of RNA was extracted from the breast muscle samples of chicken with the help of TRIZOL reagent (Invitrogen, Life Technologies, Carlsbad, CA) as per manufacturer’s instructions. The concentration and the purity of RNA was checked at the absorbance of 260/280 nm by using Nano Drop Spectrophotometer (ND 2000, Thermo Scientific Ltd). 2 μg of total RNA was used to synthesize first-strand cDNA using Oligo dT primers and reverse transcriptase M-MLV (TAKARA BIO INC.) according to manufacturer’s instructions. Thereafter, synthesized cDNA was stored at − 20 °C.

Based on known broiler sequences, specific primers were designed with Primer Premier Software (Table 2). General PCR was first performed to confirm specificity of primers. The quantitative real-time PCR was performed with few alterations on ABI Prism 7300 Detection System (Applied Biosystems, USA) (Gan et al. 2013). For performing the reactions, a 25 μL reaction mixture was made from 12.5 μL of 2 × SYBR Green I PCR Master Mix (TaKaRa BIO INC), 10 μL cDNA, each primer 1 μL (10 μM), and PCR-grade water 0.5 μL. qRT-PCR program contained a step of 95 °C for 30 s, followed by 40 cycles of 5 s at 95 °C and 31 s at 60 °C. A dissociation curve was performed per plate, which confirmed the production of a single product. The no-template control was considered as the negative control. The relative concentration of mRNA was obtained with the Δ cycle threshold (ΔCt) method as described in the applied biosystems protocol using *GAPDH* as a reference gene. Results thus obtained were applied to each gene through the calculation of the expression 2^−ΔΔCt^.

**Table 2:** Primers used for real-time PCR

Target gene	Accession no.	Primer sequence (5′–3′)	Product (bp)
*GAPDH*	K01458	Forward: TGAAAGTCGGAGTCAACGGAT Reverse: ACGCTCCTGGAAGATAGTGAT	230
*GPx1*	HM590226	Forward: AACCAATTCGGGCACCAG Reverse: CCGTTCACCTCGCACTTCTC	122
*GPx4*	AF498316	Forward: CATCACCAACGTGGCGTCCAA Reverse: GCAGCCCCTTCTCAGCGTATC	92
*HSP70*	AY288298	Forward: AGCGTAACACCACCATTCC Reverse: TGGCTCCCACCCTATCTC	372
*HSP60*	NM001012916.1	Forward: AGCCAAAGGGCAGAAATG Reverse: TACAGCAACAACCTGAAGACC	115
*HSP90*	NM0010149164	Forward: TCCTGTCCTGGCTTTAGTTT Reverse: AGGTGGCATCTCCTCGGT-3	162
*SelP*	NM_001031609	Forward: GAGGGACTGGTCAACATCTCATACG Reverse: GGGAAGACCCAGGTGGTACACT	216

### Statistical analysis

Statistical analysis of the data was carried out through statistical software package SPSS (2008, ver. 17.0). The obtained results were shown as mean ± SE. Treatment means were separated by using LSD method and the P < 0.05 was considered as statistically significant.

## Results

### Effect of selenium and vitamin-E supplementation on MDA release

**Fig. 2:**
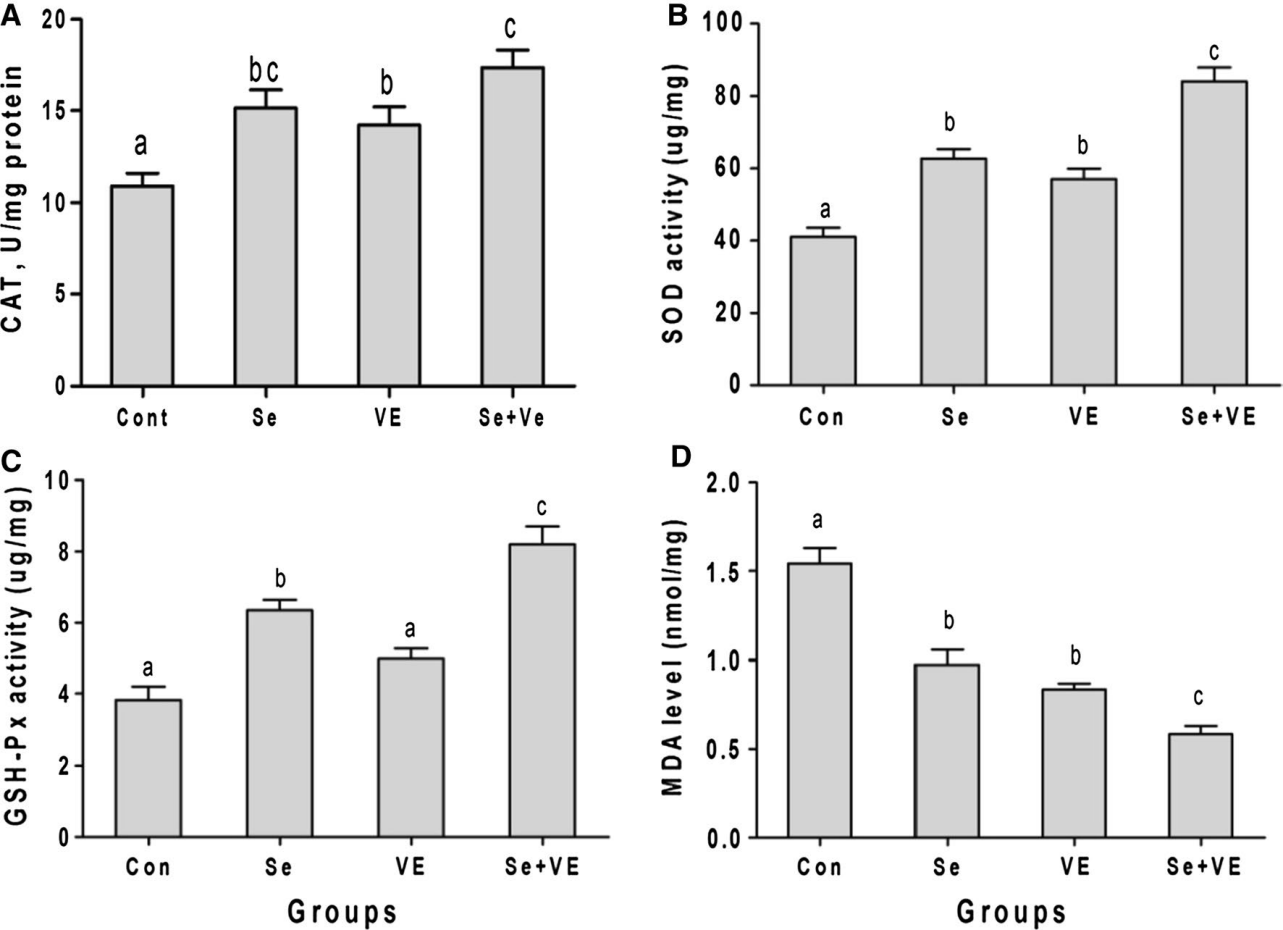
Effect of Se, VE and Se + VE on antioxidant activity **A** CAT, **B** SOD, **C** GSH-Px, and **D** MDA in breast muscles of heat stressed broiler chickens. Mean values with their standard errors. The values with unlike superscript letters (a, b, c) in the graph are different (P < 0.05)

The results of the oxidation of lipids in chest meat from different groups supplemented with or without VE and Se are shown in Fig. 2. All the treatment groups showed significantly (*P *< 0.05) reduced MDA level of breast muscle as compared with control. However, MDA level of the group Se + VE was significantly less than the Se or VE groups. Furthermore, no any significant difference was observed between the groups Se and VE.

### Antioxidant enzyme activities (AEA) in breast muscle

Se and Se + VE supplemented diets significantly (*P *< 0.05) increased the GSH-Px activity in breast muscles as compared with control and VE groups, respectively (Fig. 2). All the supplemented diets significantly increased the SOD activity as well as catalase in chicken breast meat. However, the activity was highest in Se + VE supplemented groups. Furthermore, no significant difference was observed between Se and VE treated groups.

### *Gpx1* and *Gpx4* mRNA levels in breast muscle

**Fig. 3:**
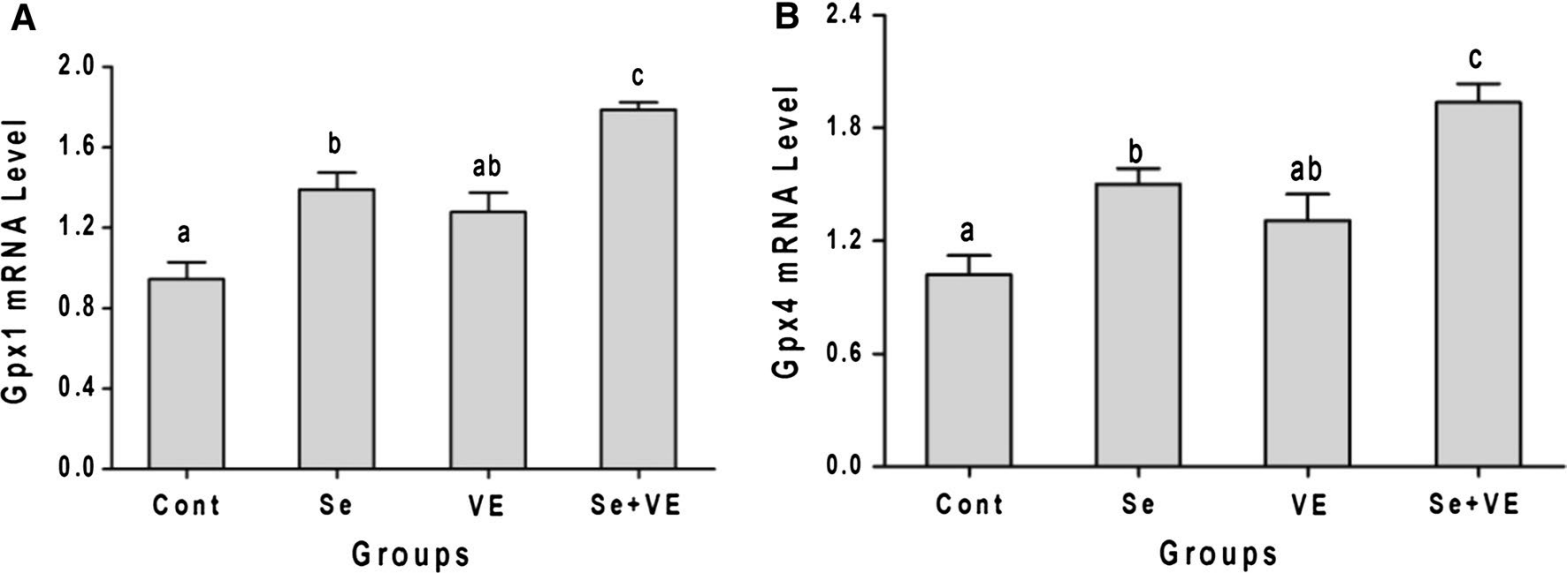
Effects of Se, VE and Se + VE on relative mRNA level of **A** Gpx1 and **B** Gpx4 in breast muscles of heat stressed broiler chickens. Mean values with their standard errors. The values with unlike superscript letters (a, b, c) in the graph were different (P < 0.05)

Tissue *Gpx1* and *Gpx4* mRNA levels obtained through real-time PCR are depicted in Fig. 3. As measured against control, no any significant (*P *< 0.05) difference in the expression levels of *Gpx1* and *Gpx4* mRNA was observed in VE group. However, significantly increased mRNA levels of *GPx1* as well as *GPx4* were found in Se and Se + VE groups. Furthermore, Se + VE group showed synergetic effect in increasing the expression levels *GPx1* and *GPx4*.

### SelP concentration and mRNA level

**Fig. 4:**
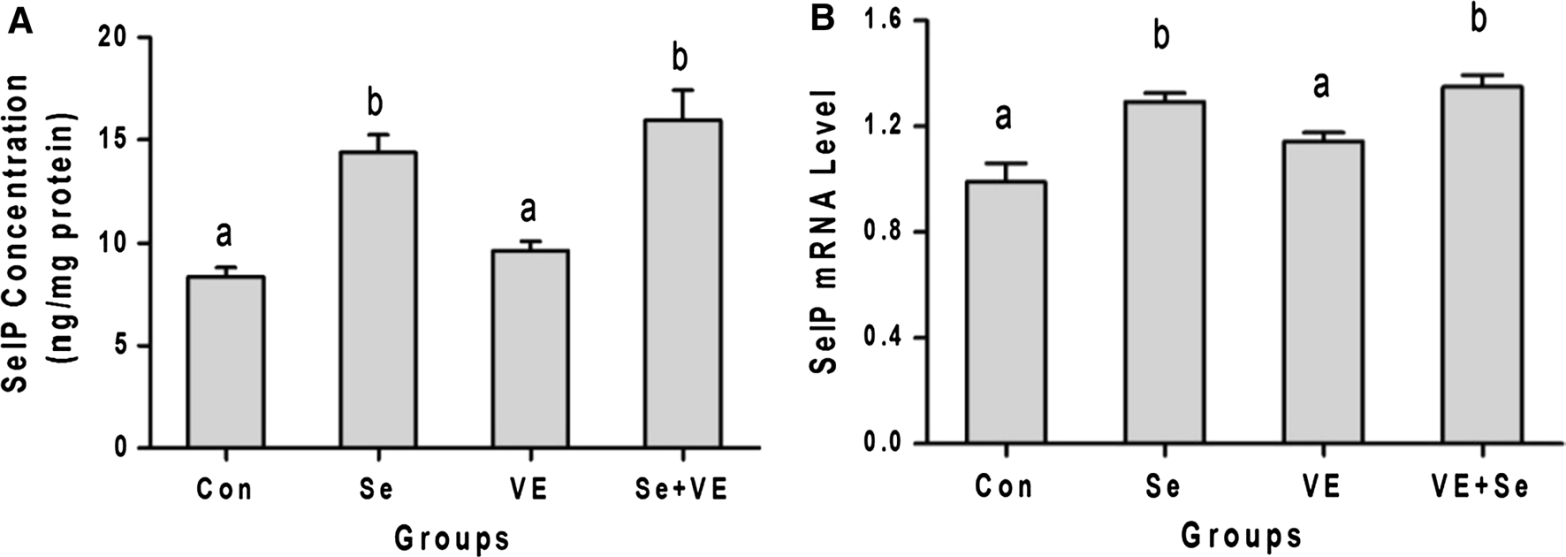
Effects of Se, VE and Se + VE on **A** SelP concentration and **B** SelP mRNA Level of breast muscles of heat stressed broiler chickens. Mean values with their standard errors. The values with unlike superscript letters (a, b, c) in the graph were different (P < 0.05)

SelP concentration and mRNA level in breast muscle of chicken is shown in Fig. 4. The Se and Se + VE dietary supplementation resulted in significantly (*P *< 0.05) increased in the levels of SelP mRNA expression as well as SelP concentration. However, VE fails to increase the concentration of SelP and mRNA level in the chicken breast muscle.

### Expression of SOD and catalase mRNA level

**Fig. 5:**
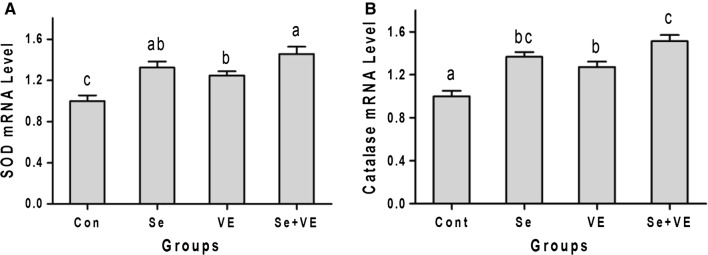
Effects of Se, VE and Se + VE on relative mRNA level of **A** SOD and **B** catalase in breast muscles of heat stressed broiler chickens. Mean values with their standard errors. The values with unlike superscript letters (a, b, c) in the graph were different (P < 0.05)

Superoxide dismutase and catalase mRNA levels of tissues obtained through real-time PCR are presented in Fig. 5. All the treatment groups exhibited significant (*P *< 0.05) increase in SOD as well as catalase mRNA levels as compared with control. However, the expression of SOD and catalase was highest in the Se + VE treatment.

### *HSPs* mRNA levels in breast meat

**Fig. 6:**
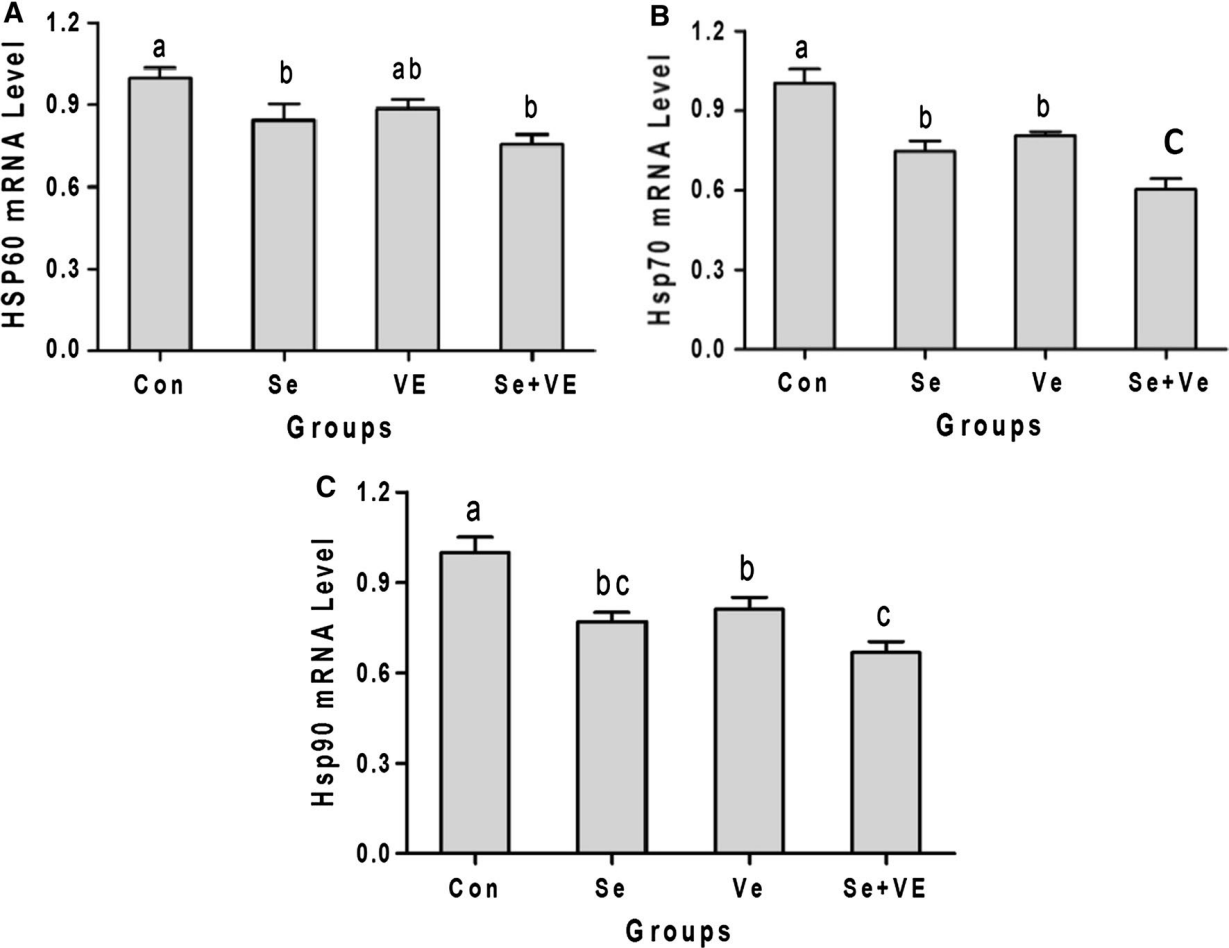
Effects of Se, VE and Se + VE on relative mRNA level of **A** HSP60 **B** *HSP70* and **C** HSP90 in breast muscles of heat stressed broiler chickens. Mean values with their standard errors. The values with unlike superscript letters (a, b, c) in the graph were different (P < 0.05)

*HSP60*, *HSP70* and *HSP90* mRNA levels in breast muscle of different groups are given in Fig. 6. As compared to control, Se and Se + VE supplemented groups significantly (*P *< 0.05) decreased the mRNA levels of *HSP90*, *HSP70* and *HSP60*. Whereas, VE diet supplementation significantly decreased the expression of *HSP90* and *HSP70* but could not obtain significant (*P *< 0.05) level of expression in case of *HSP60*. Moreover, there was non-significant difference in the decrease of *HSP60* mRNA levels in Se and VE supplemented groups.

## Discussion

Heat stress causes over-production of ROS and destroys the balance between ROS and antioxidants resulting in oxidative stress (Tan et al. 2010). Moreover, ROS are important mediators of damage to cell structures such as membranes, nucleic acids, lipids and proteins (Poli et al. 2004). Antioxidant system comprising SOD, CAT and GPx acts as a foremost line of antioxidant defense system. The MDA values obtained in this study showed a significant decrease in all dietary treatments. Combined supplementation of VE + Se in the diet proved to be the most effective inhibitor of lipid peroxidation. Furthermore, the results indicated that dietary VE and Se supplementation brought a significant increase in SOD and catalase levels of breast muscle in heat-stressed broilers. Se along with VE had a synergistic effect on the SOD, GSH-Px and catalase activity. However, the enzyme activity of GSH-Px remained depressed in case of dietary VE. These enzymes prevent the generation of free radicals and they protect the cell constituents against oxidative damage (Scott et al. 1991). In animal tissues, Se is very important element of selenoprotein enzyme GSH-Px (Skřivanová et al. 2007) and is essential for its proper functioning. Vitamin E is also a major lipid-soluble antioxidant and is known as the first defense line against cellular damage resulting from membrane phospholipid peroxidation. MDA, the most abundantly found lipid peroxide and oxidative stress indicator, thus indirectly represents the cell or tissue damage (Gan et al. 2014; Parveen et al. 2014). Earlier studies reported that combined Se and VE supplementation resulted in a reduction in the MDA level by ameliorating the antioxidant capacity of SOD and GPx of the skeletal muscle in chickens reared under heat stress (Harsini et al. 2012) which is in agreement with the findings of this study. Furthermore, MDA content in breast meat decreased by both selenium and VE supplementation under heat stress condition (Habibian et al. 2016).

The Se performs its biological functions mainly through selenoproteins, as Se is mainly incorporated on the selenoprotein active sites (Jang et al. 2014). Selenoproteins like GPx1, GPx4 and SelP play vital role in a variety of biological processes by involving in antioxidant defense system. A significant increase was observed in tissue GPx1, GPx4 and SelP mRNA level when broilers were fed Se and Se + VE supplemented diet; however, no effect on these indices was recorded in broilers given VE supplemented diet. Gpx1 is the widely present glutathione peroxidase isoforms (Bermano et al. 1995). In the presence of GSH it catalyzes the lipid hydroperoxides conversion into hydroxyl acids, thus removes the lipid peroxides (Banu et al. 2009). In addition, GPx4 is the only antioxidant selenoprotein which directly reduces phospholipid hydroperoxides and lipoproteins in the membranes (Sneddon et al. 2003). As previously reported that selenoprotein affects the Se distribution of whole body and works as antioxidant (Dokladny et al. 2006). Moreover, selenium supplementation in diet significantly increased the level of *GPx1* mRNA in piglets tissues reared under hot summer conditions. An in vitro study showed that addition of Se in the cell culture medium of caco-2 significantly increased the mRNA level expression of the *GPx1*, *TrxR1*, and *SelP* (Yavuz et al. 2004). Previous research reports have proved that over expression of *GPx1* can protect cells from damage caused by hydrogen peroxides, lipid hydroperoxides as well as redox cycling drugs like paraquat (Kelner et al. 1995). In the cell culture models and genetic mouse models, *GPx*-*1* over expression has been found associated with enhanced protection from oxidative stress (Weiss et al. 2001). A previous study demonstrated that dietary Se protected the chickens from muscular Se deficiency disease such as exudative diathesis (ED) under oxidative stress by up regulating the mRNA levels of *GPx1* and *GPx4* (Huang et al. 2011). These results indicate that Se supplementation improves the antioxidant capacity of broiler chicken by decreasing the oxidative stress and lipid peroxidation by up-regulating the selenoproteins mRNA level.

In the present study, a significant decline in the mRNA level of *HSP90*, *HSP70* and *HSP60* was found in the chicken breast meat in each treatment group under heat stress conditions. Previous studies in poultry also showed that increased heat stress resulted in the enhanced production of *HSPs* (Mahmoud et al. 2003). On other hand, exogenous supplementation with antioxidants has shown to interfere with this adaptation. The maximum effects of reducing the expression of mRNA level of *HSP70* and *HSP27* resulted due to synergetic effect of Se with probiotics in pigs reared under the natural heat stress condition (Gan et al. 2013). Treatment with α-tocopherol acetate during dry period of crossbred cows resulted in reduced oxidative stress and mRNA levels of *HSP70* (Aggarwal and AshutoshG 2013). Combination of vitamin C and E supplemented diet decreased the mRNA levels of *HSPs* in brain and ovary of Japanese quail under heat stress conditions (Sahin et al. 2009). Moreover, dietary antioxidant vitamins suppressed the expression of *HSP70* by eliminating ROS and stabilizing antioxidant status of birds in summer season (Jang et al. 2014).

Heat stress generates oxidative stress, which has been recognized as a key factor in the mediation of *HSPs* induction. In this study, the broilers fed diets supplemented with Se and VE showed lower expression of mRNA levels of *HSPs* in the breast meat. These results are in lined with previous study which reported that dietary vitamin E significantly decreased the mRNA expression of *HSP70* (Jang et al. 2014). It is likely that Se or VE diet supplementation may restrict the expression of *HSPs* by increasing the activities of SOD and subsequent removal of ROS. Recently, it has been proposed that the lipid composition and the architecture of membranes act as membrane censors and modulate *HSPs* response through the HSF-1. Therefore, the up regulation of heat shock proteins in stressed birds may be due to the damages of oxidative stress in the muscle cells. Furthermore, changes in *HSP70* may be an indication of cellular damage within the intestines (Dokladny et al. 2006). Heat stress, being a promoter of oxidative stress, increases the generation of ROS and creates a redox imbalance. The cellular damage thus caused by ROS accumulation is considered as a key factor in the activation of *HSP* genes. Cells when subjected to heat stress with increased lipid peroxidation accumulate HSP70, which might work as a tissue biomarker for potential damage caused by stress (Banu et al. 2009). Thus, the damage caused by a strong stress to the organism results in the high expression levels of constitutive and inducible *HSP70*. Besides that, the enhanced activity of SOD and catalase is thought to scavenge free radicals, which restrict the expression of HSP proteins and thus improve the cell survival.

In this study, the results came out with a significant decrease in the *HSPs* mRNA levels in the breast muscle of chicken in each treatment group as compared to control. It is noteworthy that the maximum decrease in the expression of *HSPs* could be due to combined treatment of Se and VE. The decreased expression of *HSP90*, *HSP70* and *HSP60* mRNA levels in current study may be due to the increased tissue Se and VE concentration, which in turn increased the antioxidant capacity and enhanced the *GPx1* and *GPx4* mRNA levels, which may successfully eliminate most of the ROS. In accordance with the findings of this study, the results of earlier study also reported that the addition of selenium-enriched probiotics in diet significantly decreased the expression of *HSP70* and *HSP27* in the tissues of piglets (Gan et al. 2013). This study clearly demonstrates that the Se + VE supplementation in broiler feed can enhance the endogenous antioxidant defense system by suppressing the lipid oxidation and by regulating the heat shock proteins. Hence, concluded that Se + VE is a suitable dietary supplement for broilers to ameliorate the negative effects of summer heat stress conditions.

## Abbreviations

Se: selenium
VE: vitamin E
HSP: heat shock protein
SOD: superoxide dismutase
GPx: glutathione peroxidase
MDA: malondialdehyde
ROS: reactive oxygen species
SelP: selenoprotein P
